# Colored Gingiva Composite Used for the Rehabilitation of Gingiva Recessions and Non-Carious Cervical Lesions

**DOI:** 10.3390/dj5040033

**Published:** 2017-11-20

**Authors:** Amit Paryag, Jenai Lowe, Reisha Rafeek

**Affiliations:** School of Dentistry, Faculty of Medical Sciences, The University of the West Indies, St. Augustine, Trinidad and Tobago; a.paryag@gmail.com (A.P.); jlowebpp@gmail.com (J.L.)

**Keywords:** gingival defect, gingiva-colored composite, rehabilitation

## Abstract

In this case of extensive gingival recession (Miller’s Class II) and mild physiologic pigmentation, an alternative method for the placement of Amaris Gingiva-Colored Composite was used to produce a non-invasive, aesthetic result acceptable to the patient. In restoring the defects in the entire maxilla of this patient, the opaquers were not mixed, but applied individually, directly to various areas of the teeth. Multiple opaquers were placed in a pattern mimicking the coloring of the patient’s gingiva. These were then covered with the base shade for a more aesthetic result to the patient’s satisfaction. The restorations resulting from the alternative method were highly aesthetic and at an eleven (11) month review showed no signs of failure giving rise to the conclusion that this method proposed for application of Amaris Gingiva-Colored Composite is viable for producing highly-aesthetic restorations in cases of gingival recession.

## 1. Introduction

Pink colored composites have been proposed as a viable option in the restoration of various presentations of gingival recession defects [1]. Gingival colored composites can be used to provide an aesthetic alternative [2] to surgical treatment options [3] commonly used in management of these lesions.

The method proposed by the manufacturer leads to an aesthetic solution by mixing and placement of the various opaquer shades in combination with the base nature shade composite provided, to create a restoration that blends harmoniously with the gingiva [4].

Challenges may, however, arise when dealing with extensive recession defects, such as Miller’s Class II and over, or in cases of physiologic pigmentation of the gingiva. In such cases, matching the gingival color using the standard opaquers, or blends of the opaquers may not adequately produce an aesthetic match.

This clinical report outlines an alternative method for the placement of pink colored composite in a case of extensive gingival recession (Miller’s Class II) and mild physiologic pigmentation to produce a non-invasive, aesthetic result acceptable to the patient.

Several factors influence the color of the gingival tissues including vascular supply, degree of keratinization, thickness of the epithelium and physiologic pigmentation [5]. This may change with the occurrence of disease, becoming dark red, bluish red, magenta, or deep blue in chronic inflammation; or bright red during acute inflammation. The extent of the disease process can also affect the color of the gingiva and such color changes may range through the attached gingiva or through the marginal gingiva to the mucogingival junction or through the alveolar mucosa [5].

The many variations in color [6,7] and the disparity in development of gingival aesthetics as compared to “white aesthetics” for teeth, leads to a persistent difficulty in matching the gingival color of prostheses to soft tissues [8]. This difficulty was found by the authors of this paper in previous cases [1] in attempting to mimic gingival colors and patterns when using gingiva-colored composite, particularly for lesions which extended from the cervical region toward the attached gingiva.

This may be justifiably so since two basic color zones, one comprising the attached and marginal gingiva and the other comprising the adjacent alveolar mucosa have been noted in oral cavities of most people [9]. Additionally, when using the Munsell color system, gingival color has been noted to vary with the position of the papillary, marginal and attached gingiva [10].

## 2. Case Report

A 45 year old male patient with a clear medical history presented to the author’s private practice. He reported being an irregular dental attender and flossed daily and brushed with a medium toothbrush scrub technique. His intra-oral findings revealed extensive recession defects (Miller’s Class II) in both the maxilla and mandible, Class I Molar relationship, Class III Incisor relationship and bilateral posterior crossbite.

**Figure 1:**
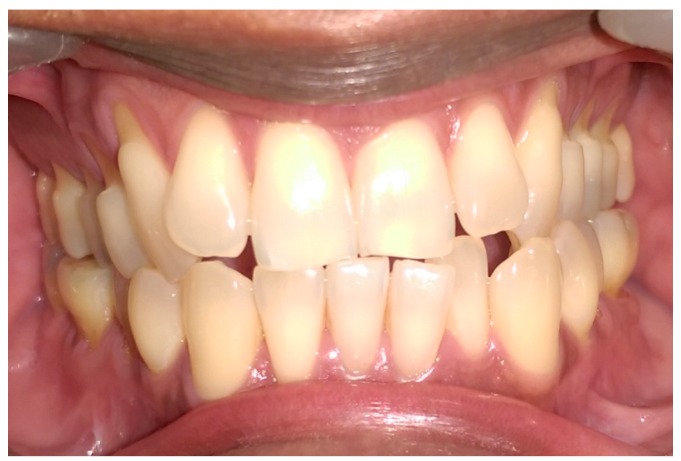
Preoperative anterior view.

The pre-operative intra-oral appearance is shown in Figure 1 and treatment options given are presented in Table 1.

Having presented the patient in this case with information regarding how his occlusal pattern, brushing habits, and other factors could be contributing to the observed recession defects, the patient opted to have only the maxillary arch restored with a gingiva-colored composite. The patient chose to proceed with Option 4 for treatment.

**Figure 2:**
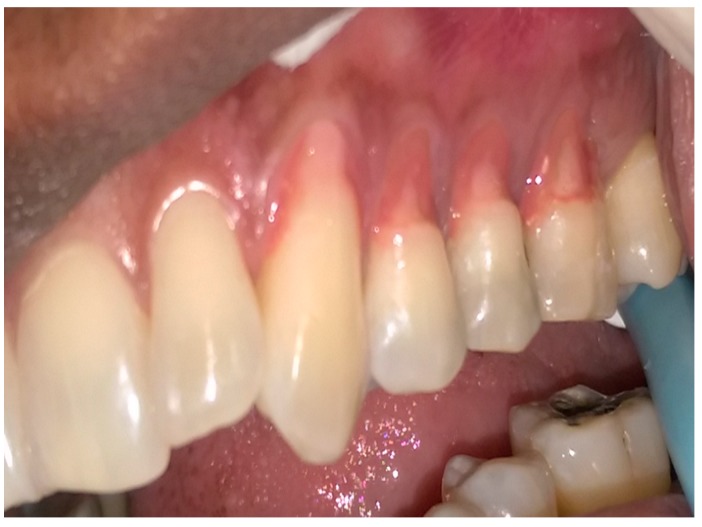
Initial placement of the dark opaque.

**Figure 3:**
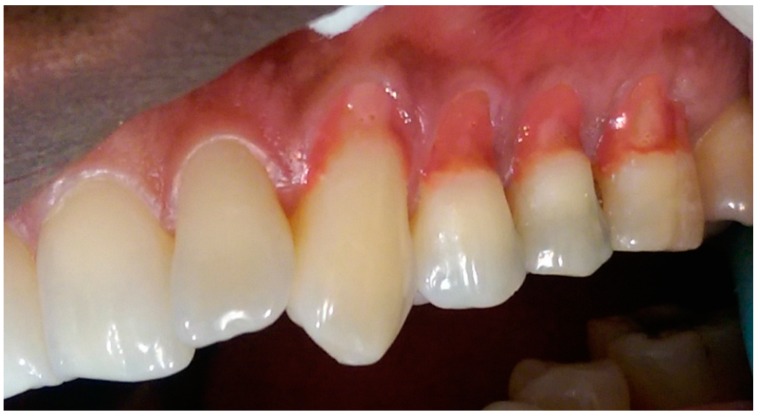
Light and white opaque placement.

**Figure 4:**
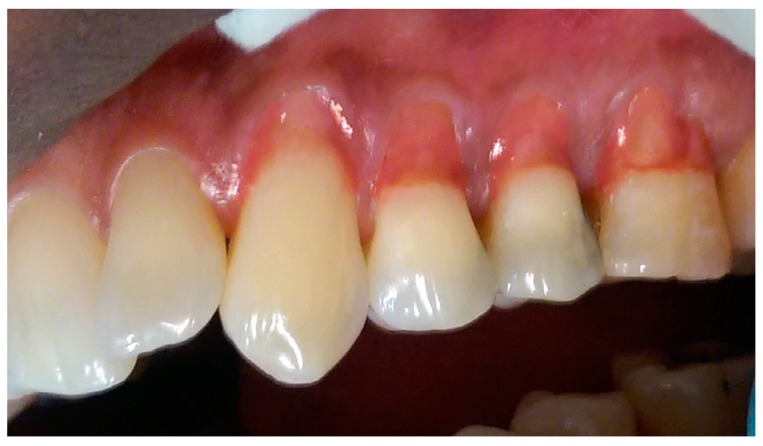
Blending of dark, white and light opaquers.

**Figure 5:**
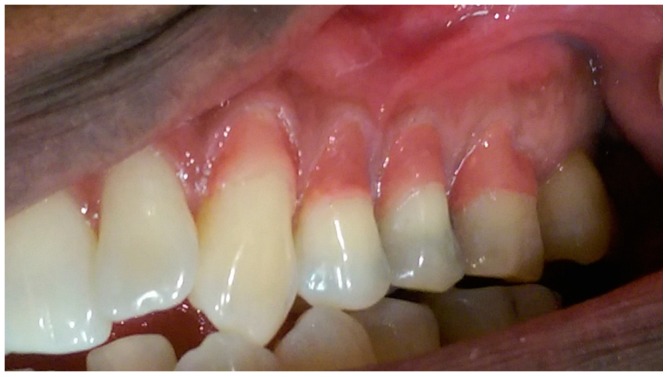
Opaquer placement for characterization of gingiva.

**Figure 6:**
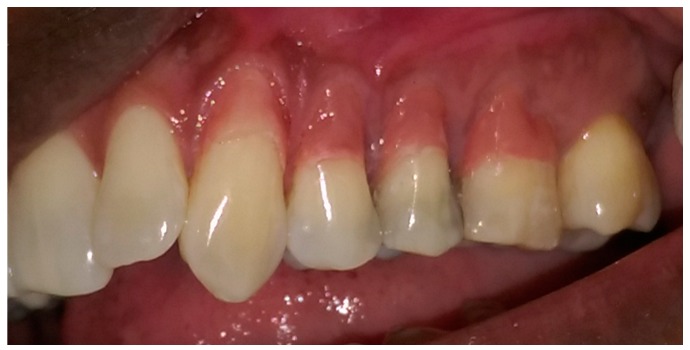
Placement and sculpting of nature shade composite.

**Figure 7:**
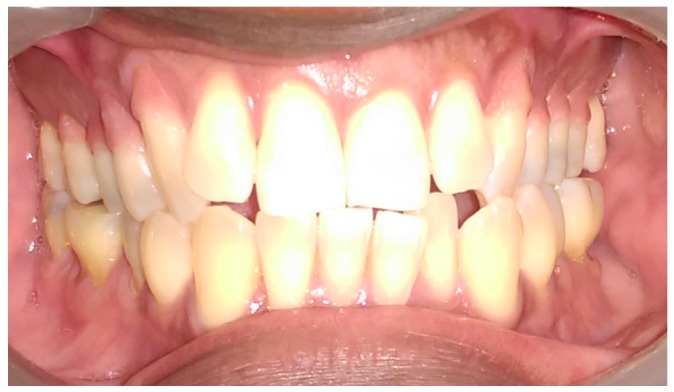
Final appearance of restored lesions.

Table 2 describes the sequence technique for placing the Amaris Gingiva-Colored Composite and Figure 2, Figure 3, Figure 4, Figure 5, Figure 6 and Figure 7 illustrates the clinical steps in which the gingiva-colored composite was placed to maximize the aesthetic effect.

During the initial evaluation the patient had a Basic Periodontal Examination (BPE) and recoded BPE codes of 1 in all sextants, which means bleeding on probing. The plaque score was less than 20% with probing depths of 2–3 mm on all teeth present. The patient exhibited generalized Miller’s Class II recession defects (marginal recession extending to or beyond the mucogingival junction with no loss of interdental bone or soft-tissue) and between 3–4 mm recession on teeth in left and right posterior sextants. There was no complaint of sensitivity. Given the initial findings and the observed occlusal discrepancies, it was determined that the patient’s etiology of recession was a combination of abnormal cervical forces secondary to occlusal discrepancies with a secondary etiology of improper brushing technique. The patient’s previously-established method of brushing was a scrub technique using a medium toothbrush. He was instructed to use a soft toothbrush and Modified Stillman Technique since there were extensive recession defects present. Six-month review periods were established to review the oral hygiene, progression of the recession, as well as the stability of the restorations done.

## 3. Discussion

In this particular case, the patient’s recession defects extended from the cervical margin to the attached gingiva. The color variation ranging from his papillary and cervical regions toward his attached gingiva are clearly seen in Figure 2, Figure 3, Figure 4, Figure 5, Figure 6 and Figure 7.

The material selected for the restoration of the patient was Amaris Gingiva (VOCO America, Inc., Indian Land, SC, USA). It is a methacrylate-based composite using bisphenol-A-glycidyldimethacrylate (BISGMA), urethane dimethacrylate (UDMA), and tetraethyleneglycoldimethacrylate (TEGDMA) as matrix monomers.

The manufacturer-advised method of application of individual opaquers or mixing the opaquers prior to application, to produce a matching gingival color generally gives acceptable results [1,4]. In larger defects, however, they may not adequately produce patterns mimicking those seen in the gingiva. In this case, the opaquers were applied directly to the teeth in patterns mimicking the color of the gingiva (Figure 2, Figure 3, Figure 4, Figure 5 and Figure 6).

The darker shade opaquer was placed at the margin of the defect (Figure 2) and the lighter shade opaquer over the area that would represent the most buccal prominence of the roots (Figure 3). Where necessary, they were blended directly on the tooth to produce the required color (Figure 3 and Figure 4). The color was varied from cervical to apical to match the pattern of gingival color variation from papilla to marginal and attached gingiva. Each placement of opaquer was cured prior to applying the next increment. Opaquers were also applied and cured as required for characterization across the initial opaquer placement (Figure 5). Having covered the root surface in the desired color, the natural shade gingiva was applied and sculpted to match the adjacent gingival contours (Figure 6). A cervical line was created at a uniform height and in a uniform shape to enhance the natural appearance of the final restoration (Figure 6).

The customization of opaquer placement on individual teeth to match the adjacent gingiva and the maintenance of cervical line height resulted in a natural appearance of the restorations postoperatively (Figure 6 and Figure 7).

**Figure 8:**
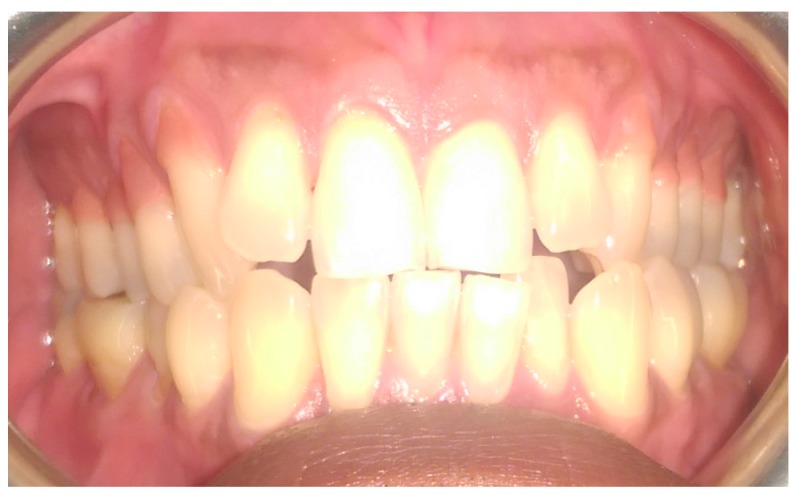
Post operative view at 11 months.

Cases such as these may also have been managed with surgical techniques, such as free gingival graft, sub-epithelial connective tissue graft, semilunar flaps, coronally-advanced flaps, and guided tissue regeneration. It is essential that, in restorative treatment, the biological width is not encroached upon as placement of restorations within this zone can lead to potential further recession [3]. The patient did not return to the surgery for eleven (11) months due to personal reasons. When he did return, it was observed that the appearance was maintained without failure of the margins or loss of color (Figure 8). There was no complaint of sensitivity on the initial presentation. No new occurrence of sensitivity was reported, nor did the patient note any increased comfort when brushing, eating, or drinking. However, further long-term follow up should occur as complications, such as chipping, fracturing, or debonding of restorations, may arise, as well as loss of color stability.

## 4. Conclusions

Several factors influence the gingival color. The many variations in gingival color, and lack of development in gingival aesthetics make matching of the color of prostheses difficult. The same applies to the placement of gingival composite in extensive lesions. The method described herein, which seeks to place the gingival opaquers in a pattern which mimics the natural gingiva, can be a viable method for cost effective, aesthetic rehabilitation of extensive gingival recession defects using gingiva-colored composite.

Given the results seen at this time, it is suggested that careful consideration should be given to observing and mapping the changes in gingival color prior to placement of Amaris Gingiva opaquers. Subsequent to this, the method for placement described and illustrated in this paper is proposed as a viable method for placement of Amaris Gingiva Composite for the restoration of extensive recession defects, to produce a cost effective, aesthetic result.

## Figures and Tables

**Table 1:** Treatment options.

Option Number	Treatment Detail
Option 1	Do nothing; monitor the dentition and, should the lesions become painful, then intervene.
Option 2	Orthodontic treatment for correction of the crossbite; surgical Intervention for correction of recession defects.
Option 3	Orthodontic treatment for correction of the crossbite; gingiva-colored composites to aid in visualizing the possible result should surgery be a future option.
Option 4	Gingiva-colored composites to restore the defects and aid in visualizing the possible result should surgery be a future option. Monitor the composites for failure and replace as necessary.

**Table 2:** Placement sequence for Amaris Gingiva-Colored Composite.

Steps	Sequence
1	The patient opted to have only the maxillary arch restored.
2	After pumicing lightly and etching the teeth using 34% phosphoric acid and rinsing, the entire segment was isolated using cotton rolls and low volume saliva ejector.
3	Clearfil Universal Bond Adhesive Resin (Kuraray Noritake Dental Inc., KURARAY AMERICA, INC. 33 Maiden Ln., Suite 600D, New York, NY 10038, USA) was placed via a microtip applicator and burnished—20 s; air dried—10 s; light cured—30 s.
4	Opaquers were applied directly to the teeth in patterns mimicking the color of the gingiva. Where necessary they were blended directly on the tooth to produce the required color.
5	Each placement of opaquer was cured prior to applying the next increment.
6	Having covered the root surface in the desired color, the nature shade gingiva was applied and sculpted to match the adjacent gingival contours.
7	A cervical line was created at a uniform height and in a uniform shape to enhance the natural appearance.
